# Supplementary material for: Outbreaks of H5N1 High Pathogenicity Avian Influenza in South Africa in 2023 Were Caused by Two Distinct Sub-Genotypes of Clade 2.3.4.4b Viruses
Source: Viruses. 2024 May 31;16(6):896. doi: 10.3390/v16060896 (PMC11209199; doi:10.3390/v16060896)
Supplement: Supplementary file 1 [file viruses-16-00896-s001.zip › viruses-3028589-supplementary materials/viruses-3028589-supplementary materials/Figure S2 a to h.pdf]

Figure S2 (a) to (h). Maximum likelihood phylogenetic trees of the eight genome segments of South African clade 2.3.4.4B H5N1 HPAI viruses and the closest relatives. Viruses sequenced in this study are in boldface. PB2- polymerase B2; PB1- polymerase 1; PA- polymerase A; HA- hemagglutinin (H5 subtype); NP- nucleocapsid protein; NA- neuraminidase; M- matrix protein, NS- non-structural protein.

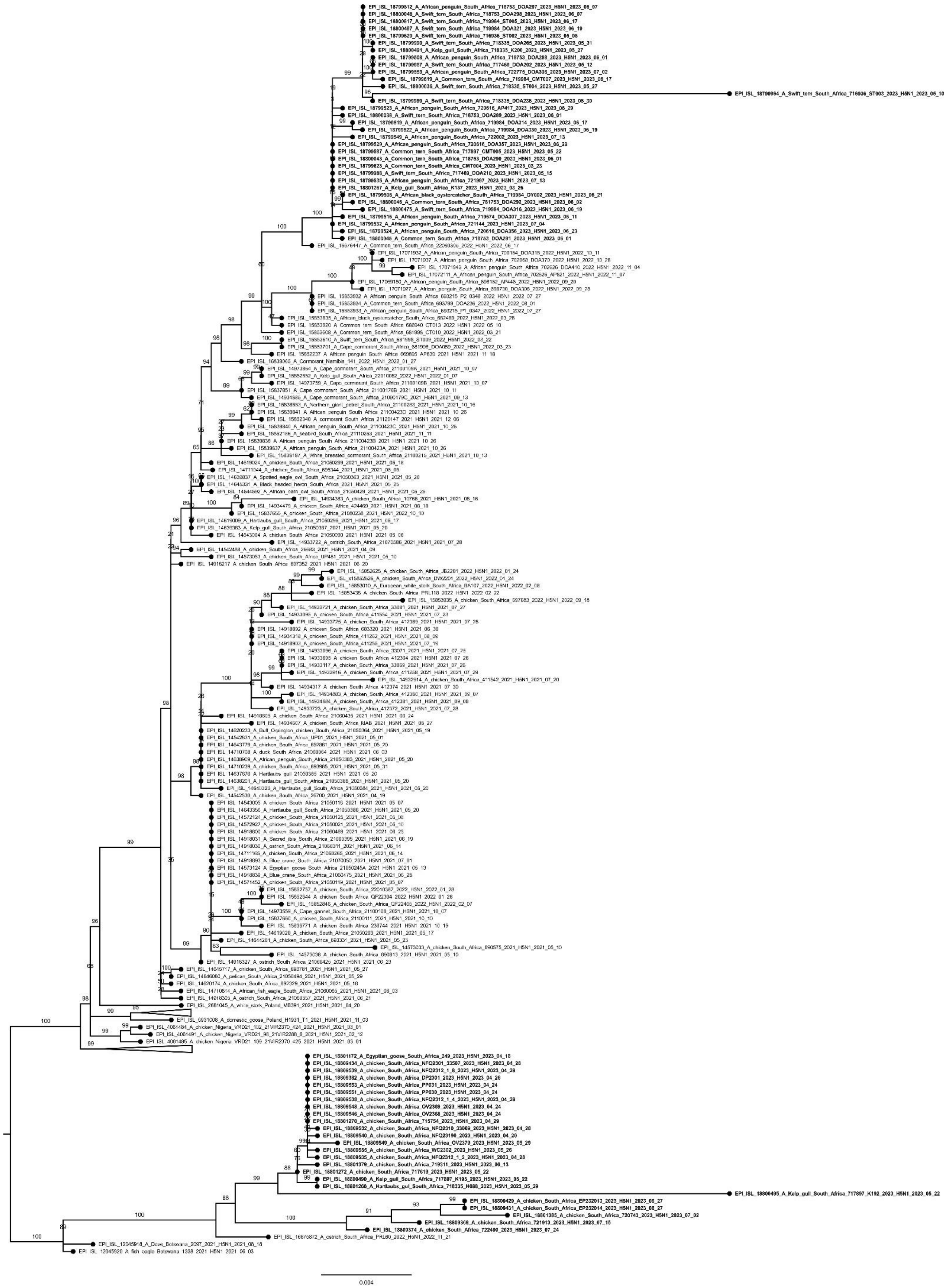

S1(a). PB2

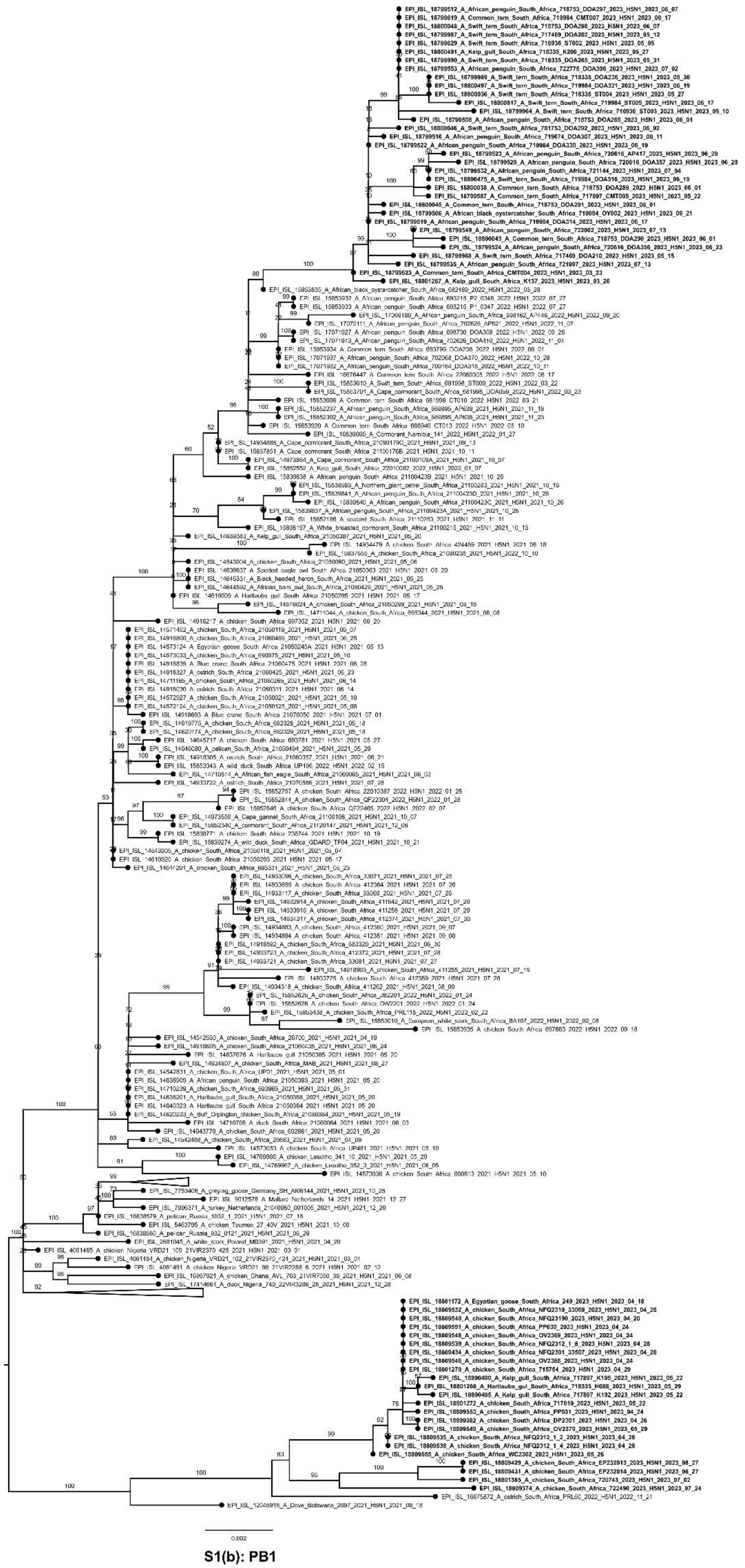

S1(b): PB1

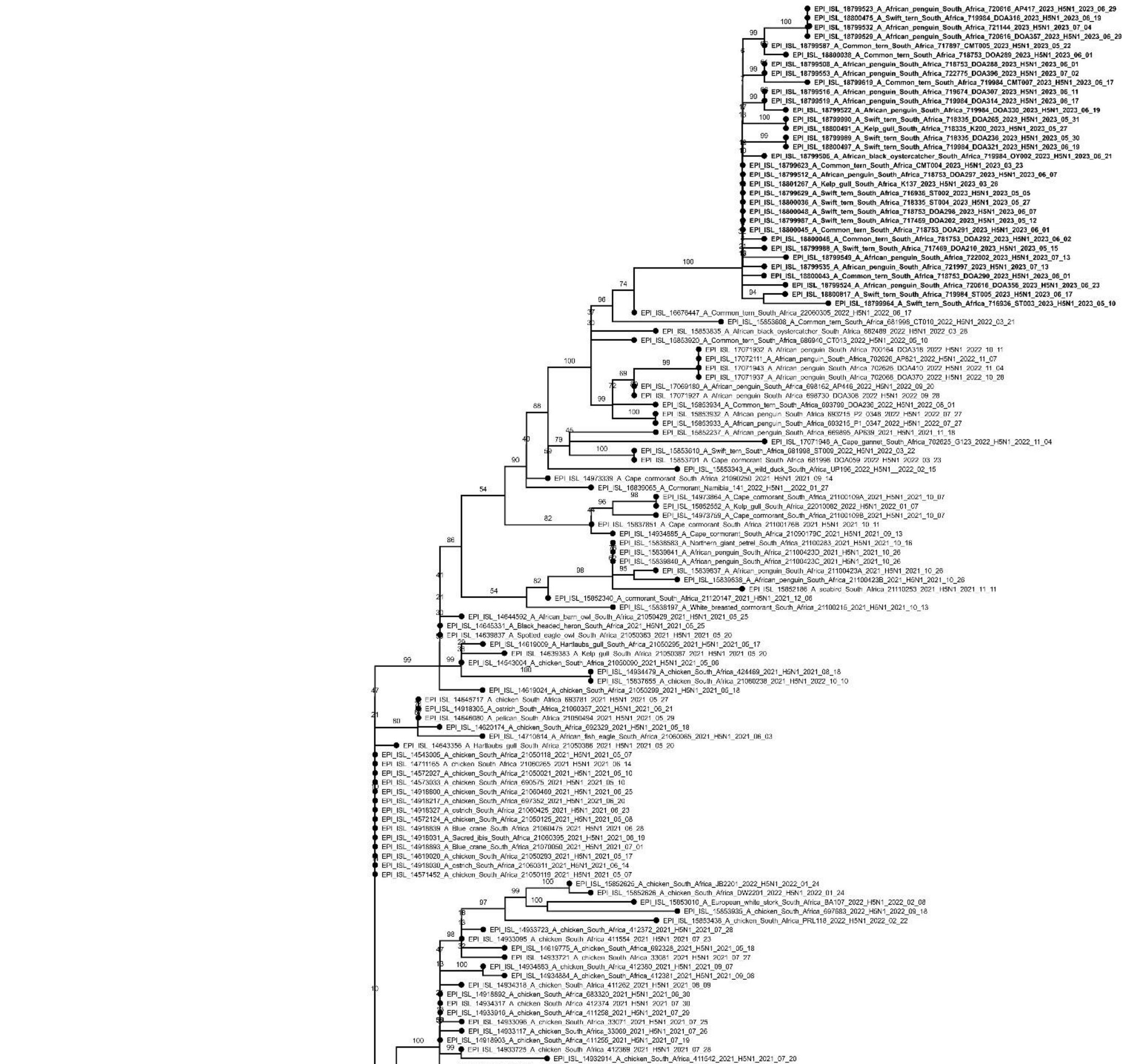

S1(c): PA

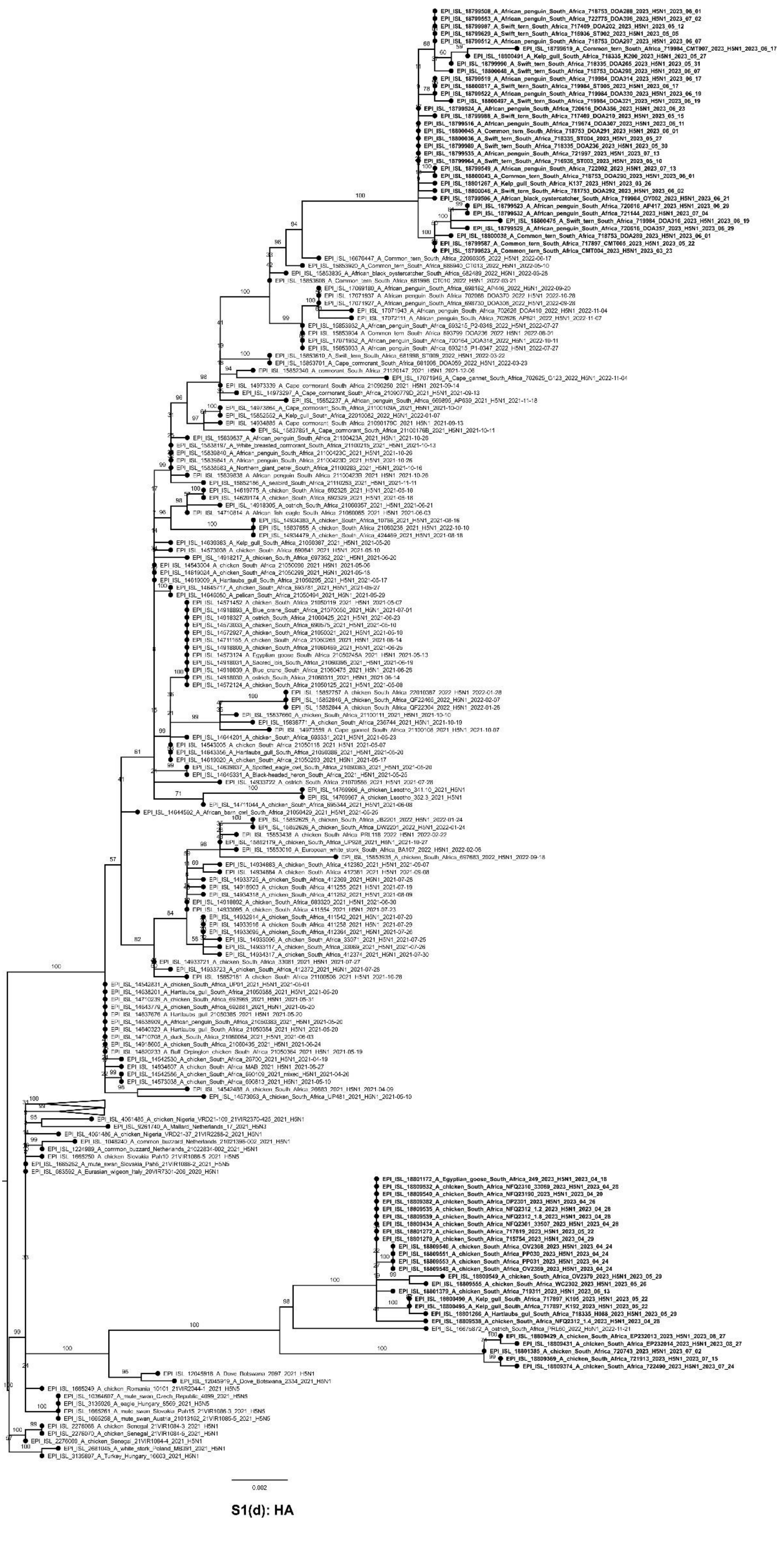

S1(d): HA

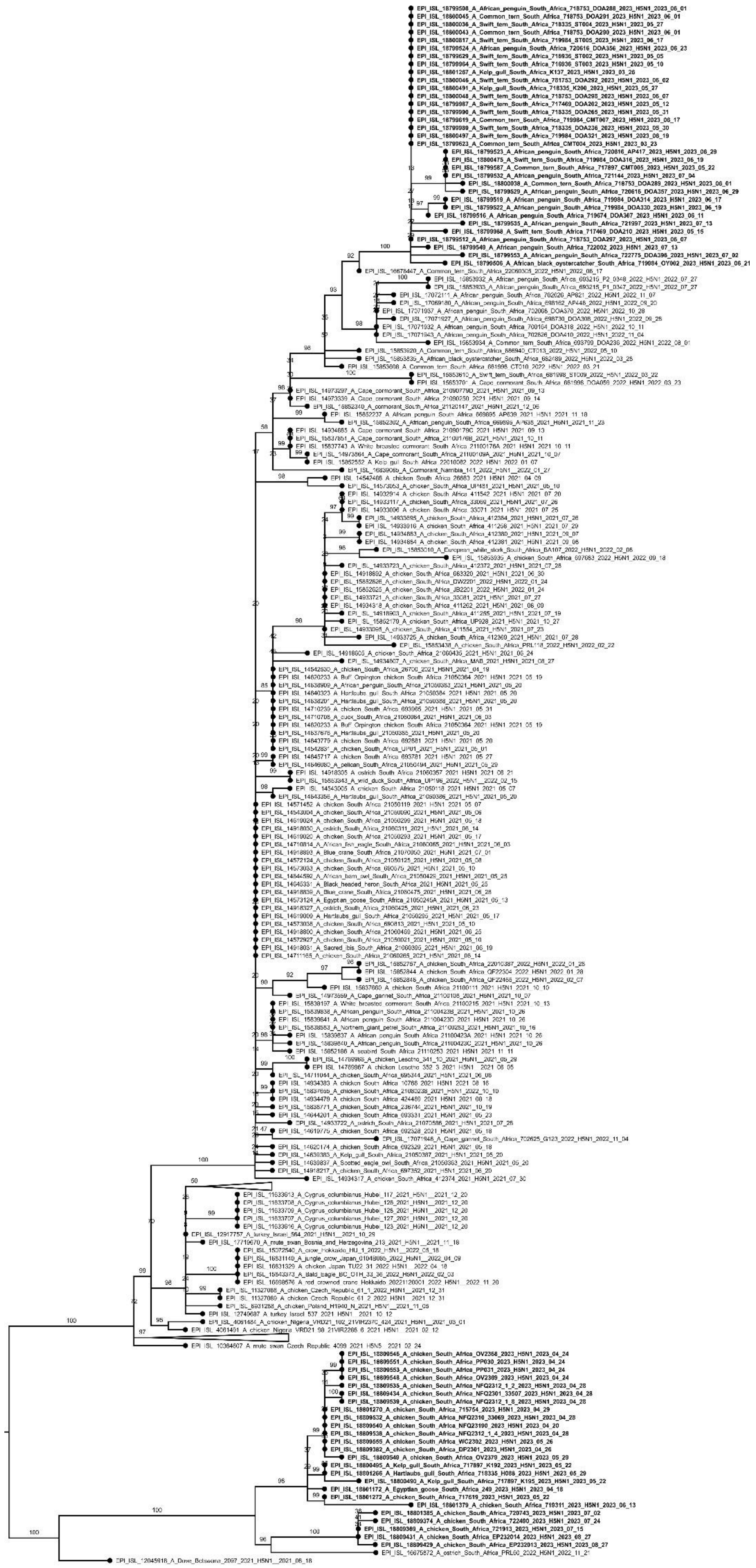

S1(e): NP

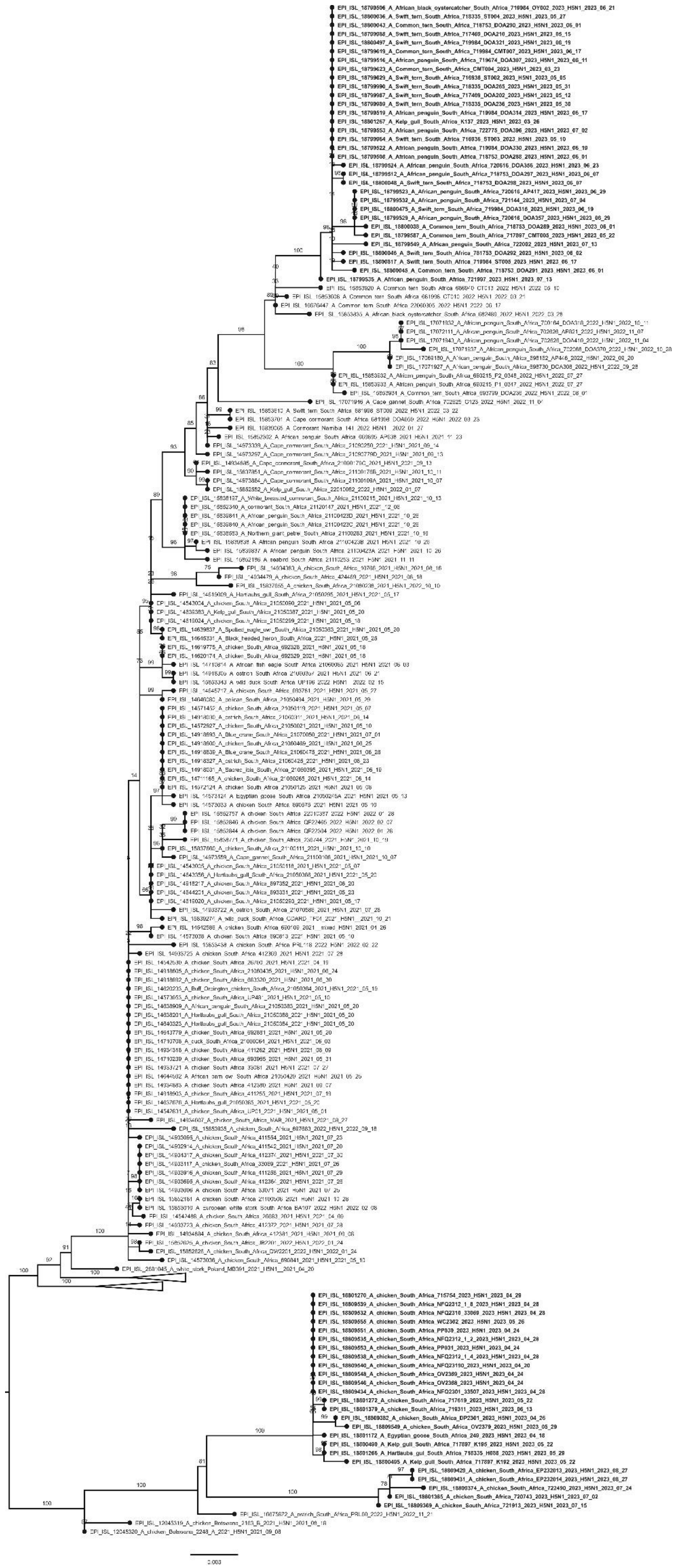

S1(f): NA

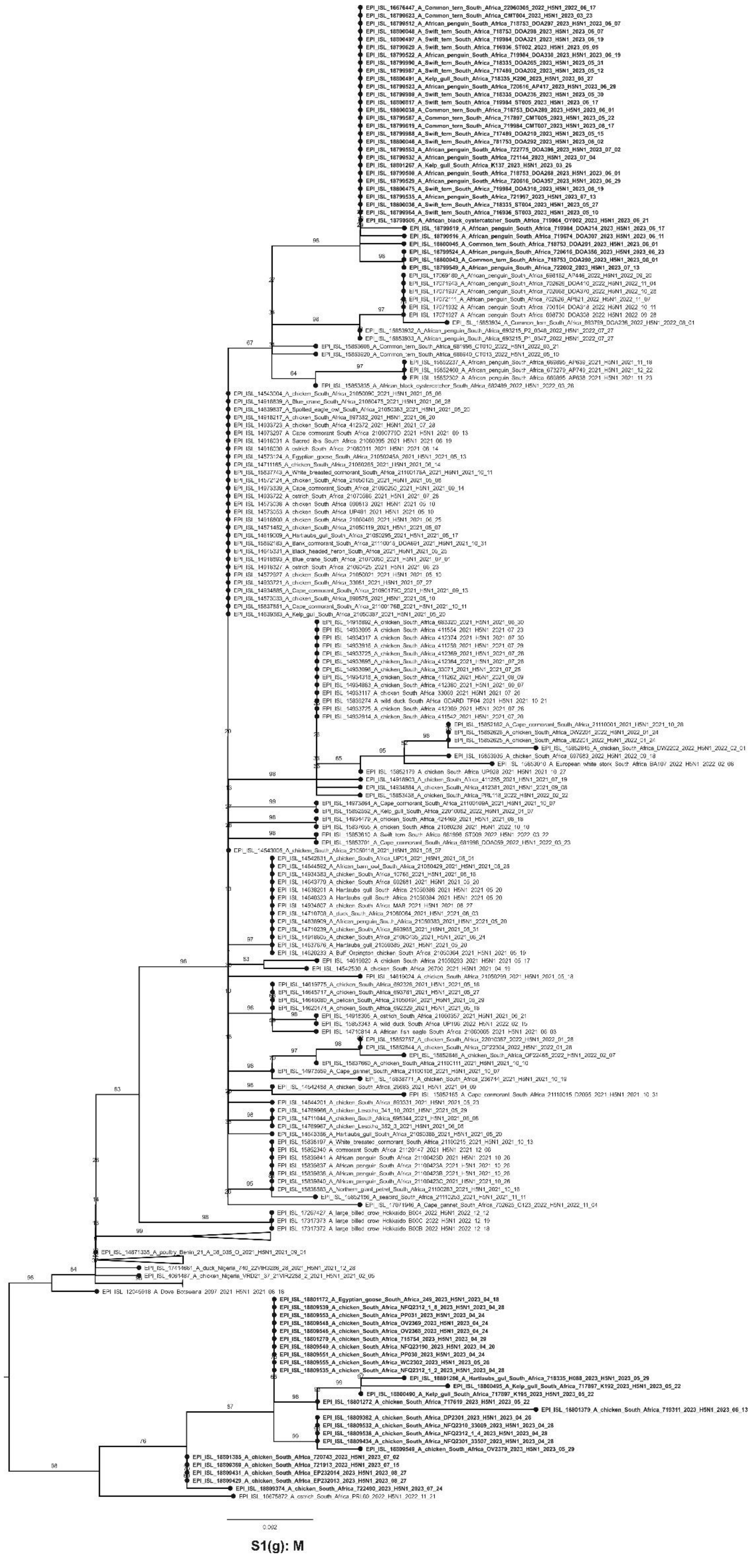

S1(g): M

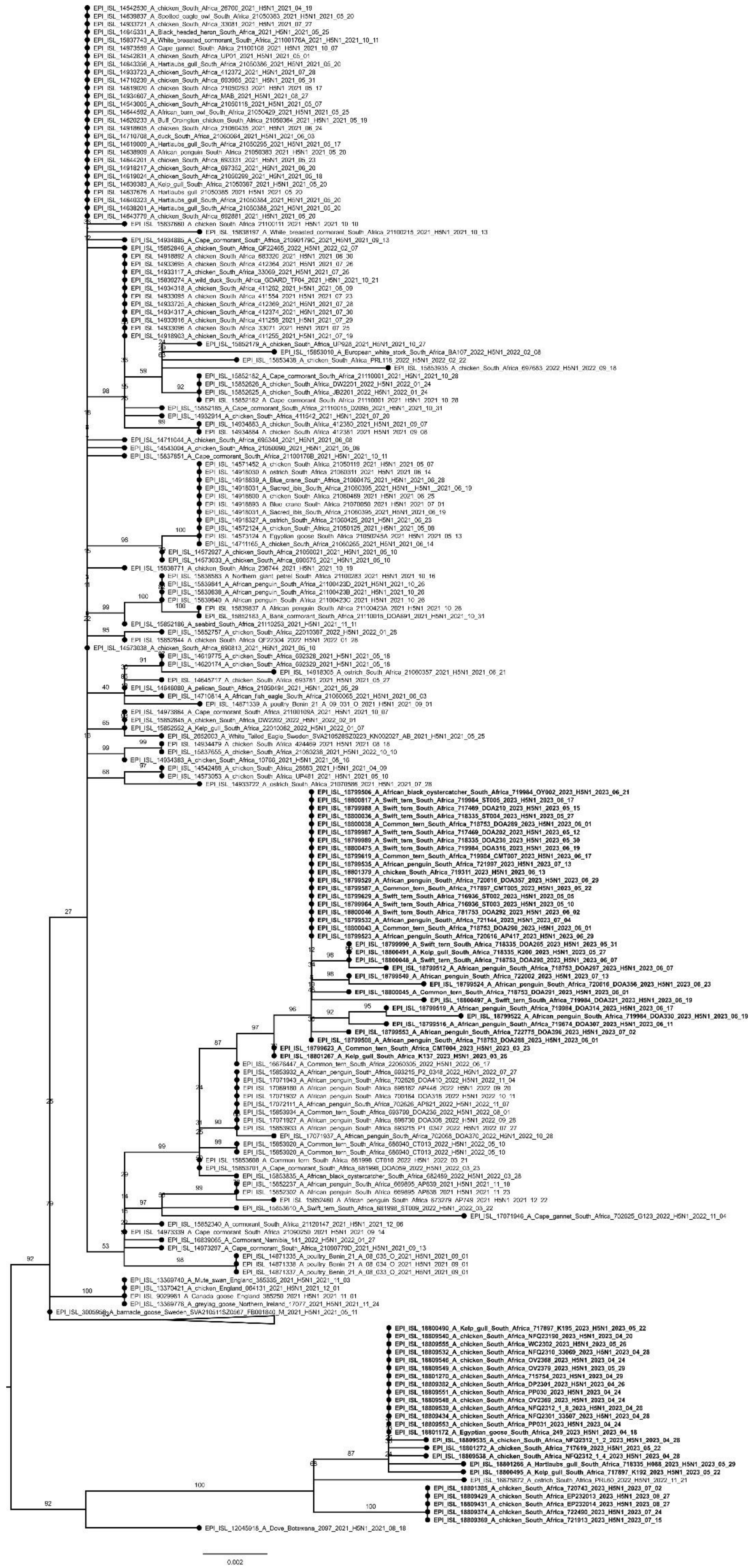

S1(h): NS
