# Supplementary material for: Outbreaks of H5N1 High Pathogenicity Avian Influenza in South Africa in 2023 Were Caused by Two Distinct Sub-Genotypes of Clade 2.3.4.4b Viruses
Source: Viruses. 2024 May 31;16(6):896. doi: 10.3390/v16060896 (PMC11209199; doi:10.3390/v16060896)
Supplement: Supplementary file 1 [file viruses-16-00896-s001.zip › viruses-3028589-supplementary materials/viruses-3028589-supplementary materials/Figure S3.pdf]

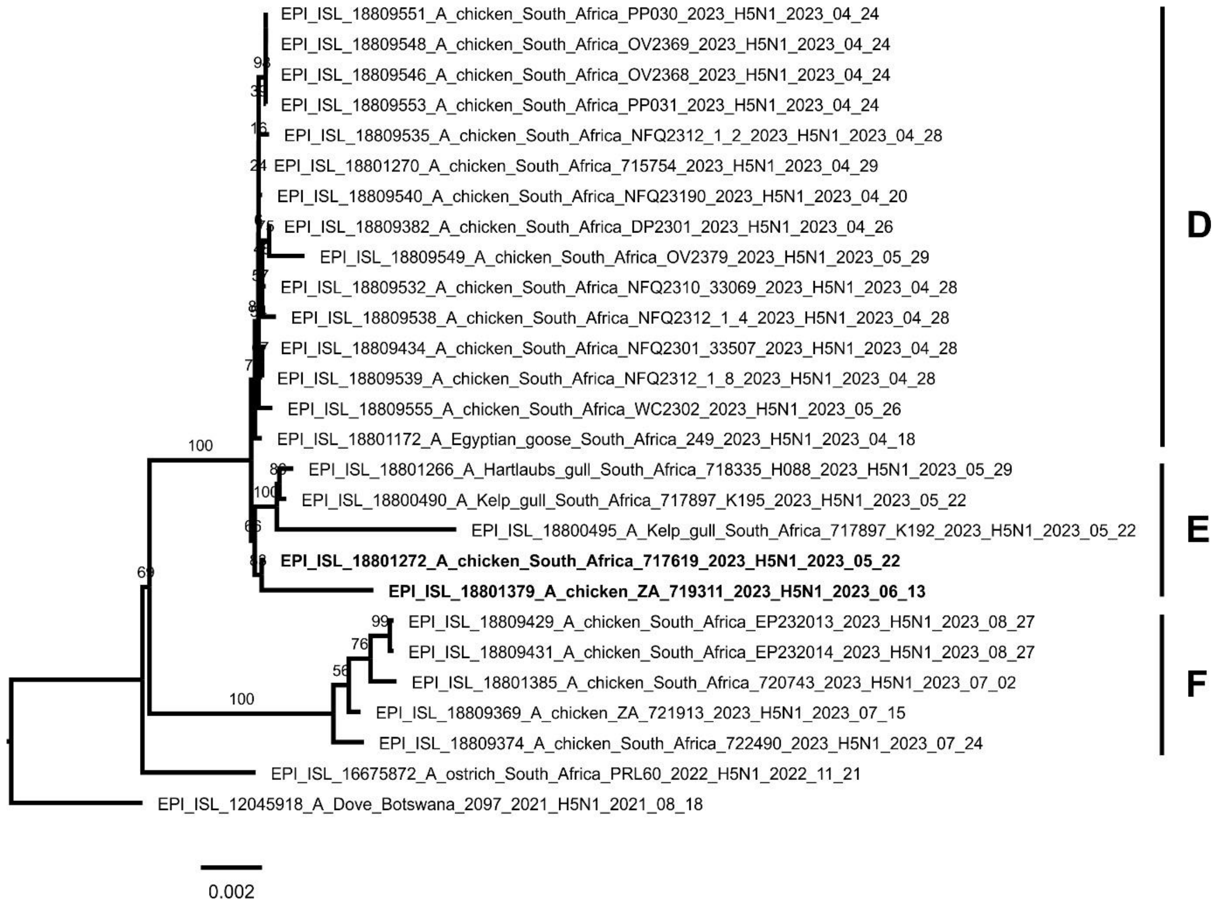

Fig. S3. Maximum likelihood phylogenetic tree of the concatenated HA, M, NA, NS, NP and PB2 genes of SA15 viruses (8766 nt); (721913 lacks a PB1 and 719311 lacks PB1 and PA). Additional sequences are in boldface.
