# Supplementary material for: Outbreaks of H5N1 High Pathogenicity Avian Influenza in South Africa in 2023 Were Caused by Two Distinct Sub-Genotypes of Clade 2.3.4.4b Viruses
Source: Viruses. 2024 May 31;16(6):896. doi: 10.3390/v16060896 (PMC11209199; doi:10.3390/v16060896)
Supplement: Supplementary file 1 [file viruses-16-00896-s001.zip › viruses-3028589-supplementary materials/viruses-3028589-supplementary materials/Table S1.pdf]

Supplemental Table 1. Monthly number of environmental fecal swab pools (n= 130 [a]) from wild ducks tested for the presence of avian influenza (AIV) and H5Nx/H7Nx subtype- specific viral RNA in 2023.

**Province [b]**	**Jan**	**Feb**	**Mar**	**Apr**	**May**	**Jun**	**Jul**	**Aug**	**Sep**	**Oct**	**Nov**	**Dec**
**Eastern Cape**			**Total: 1**<br>AIV:1<br>H5:0<br>H7:0	**Total: 2**<br>AIV:1<br>H5:0<br>H7:0								
**Gauteng**		**Total: 77**<br>AIV:57<br>H5:27<br>H7:0	**Total: 66**<br>AIV:63<br>H5:44<br>H7:0	**Total: 4**<br>AIV:3<br>H5:0<br>H7:0	**Total: 8**<br>AIV:6<br>H5:0<br>H7:0	**Total: 2**<br>AIV:0		**Total: 7**<br>AIV:2<br>H5:1<br>H7:0				
**KwaZulu-Natal**			**Total: 1**<br>AIV:0	**Total: 10**<br>AIV: 10<br>H5:2<br>H7:1	**Total: 11**<br>AIV:6<br>H5:0<br>H7:0	**Total: 8**<br>AIV:2<br>H5:1<br>H7:0	**Total: 7**<br>AIV:3<br>H5:0<br>H7:0	**Total: 7**<br>AIV:3<br>H5:2<br>H7:0	**Total: 8**<br>AIV:2<br>H5:1<br>H7:0	**Total: 7**<br>AIV:4<br>H5:0<br>H7:0	**Total: 9**<br>AIV7<br>H5:0<br>H7:0	
**Mpumalanga**			**Total: 2**<br>AIV:1<br>H5:1<br>H7:0		**Total: 3**<br>AIV:1<br>H5:0<br>H7:0	**Total: 1**<br>AIV:1<br>H5:0<br>H7:0						
**North West**		**Total: 1**<br>AIV:1<br>H5:1<br>H7:0			**Total: 4**<br>AIV:0		**Total: 16**<br>AIV:6<br>H5:0<br>H7:0	**Total: 17**<br>AIV:10<br>H5:0<br>H7:0				
**Western Cape**		**Total: 6**<br>AIV:2<br>H5:0<br>H7:0		**Total: 8**<br>AIV:8<br>H5:5<br>H7:0	**Total: 24**<br>AIV:4<br>H5:0<br>H7:0	**Total: 15**<br>AIV:11<br>H5:4<br>H7:0	**Total: 7**<br>AIV:7<br>H5:4<br>H7:0	**Total: 15**<br>AIV:7<br>H5:0<br>H7:0		**Total: 28**<br>AIV:11<br>H5:0<br>H7:1	**Total: 8**<br>AIV:2<br>H5:0<br>H7:0	
**Total**	0	**60**	**65**	**22**	**17**	**14**	**16**	**22**	**2**	**15**	**9**	

[a] 47 samples from various provinces without sampling dates are excluded from this table.

[b] No samples received from the Free State, Limpopo or Northern Cape provinces
