# Supplementary material for: Outbreaks of H5N1 High Pathogenicity Avian Influenza in South Africa in 2023 Were Caused by Two Distinct Sub-Genotypes of Clade 2.3.4.4b Viruses
Source: Viruses. 2024 May 31;16(6):896. doi: 10.3390/v16060896 (PMC11209199; doi:10.3390/v16060896)
Supplement: Supplementary file 1 [file viruses-16-00896-s001.zip › viruses-3028589-supplementary materials/viruses-3028589-supplementary materials/Table S2.pdf]

Table S2. BLAST sequence homology results for avian influenza viruses detected in commercial ostriches in 2023

	PB2	PB1	PA	HA	NP	NA	M	NS
EPI19009636<br>A/ostrich/South Africa/AI9145-P42/2023<br>(H6N2)	EPI2042329[1]<br>A/ostrich/South Africa/18090431/2018 (H11N1)[2]<br>3906.8[3]<br>2225/2280<br>(97.59%)[4]	EPI1850442<br>A/common teal/Novosibirsk region/3559k/2020 (H4N6)<br>4065.6<br>2274/2310<br>(98.48%)	EPI1877846<br>A/White-Tailed Eagle/Sweden/SVA210527SZ0358/KN002111/C-2021<br>(H5N5)<br>3853.2<br>2163/2201<br>(98.27%)	EPI1567203<br>A/wild waterfowl/Korea/F56-1/2017<br>(H6N2)<br>2700.9<br>1636/1722<br>(95.01%)	EPI2868129<br>A/duck/Zambia/UNZA-264/2021<br>(H11N6)<br>2724.9<br>1511/1529 (98%)	EPI2131862<br>A/wild bird/South Africa/ZL309/2022 (H9N2)<br>2466.4<br>1386/1411<br>(98.82%)	EPI1850431<br>A/common teal/Novosibirsk region/3556k/2020 (H3N8)<br>1781.3<br>982/991 (99.09%)	EPI2868115<br>A/duck/Zambia/UNZA-028/2020<br>(H8N4)<br>1546.8<br>859/870 (98.74%)
EPI17851396<br>A/ostrich/South Africa/761940/2023 (H12N2)	EPI2868132<br>A/duck/Zambia/UNZA-264/2021<br>(H11N6)<br>3989.9<br>2258/2307<br>(97.88%)	EPI2868114<br>A/duck/Zambia/UNZA-028/2020<br>(H8N4)<br>4037.9<br>2270/2311<br>(98.23%)	EPI1849905<br>A/mallard/Novosibirsk region/3445k/2020 (H1N1)<br>588.4<br>353/370 (95.41%)	EPI1640115<br>A/mallard/Novosibirsk region/999k/2018<br>(H12N5)<br>1046.3<br>610/632 (96.52%)	EPI1968922<br>A/goose/France/21P014207/2021<br>(H5N1)<br>2641.8<br>1496/1529<br>(97.84%)	EPI2043196<br>A/chicken/Kenya/440560/2021<br>(H9N2)<br>483.1<br>263/264 (99.62%)	EPI2868133<br>A/duck/Zambia/UNZA-264/2021<br>(H11N6)<br>1620.6<br>955/993 (96.17%)	EPI2044469<br>A/ostrich/South Africa/070595/2020 (H7N1)<br>1537.5<br>868/886 (97.98%)

[1]Accession number
[2]Strain and subtype
[3]Score (Sum of alignment scores for all of the sequence segments or local alignments. The higher the score, the better the alignment)
[4]Nucleotide sequence identity
