# Supplementary material for: Outbreaks of H5N1 High Pathogenicity Avian Influenza in South Africa in 2023 Were Caused by Two Distinct Sub-Genotypes of Clade 2.3.4.4b Viruses
Source: Viruses. 2024 May 31;16(6):896. doi: 10.3390/v16060896 (PMC11209199; doi:10.3390/v16060896)
Supplement: Supplementary file 1 [file viruses-16-00896-s001.zip › viruses-3028589-supplementary materials/viruses-3028589-supplementary materials/Table S3.pdf]

Table S3. Unique markers in the translated protein sequences that emerged in coastal seabird H5N1 HPAI sub-lineage SA13 viruses.

Protein	Mutation	2021	2022	2023
**PB2**	E6K	✓ (blue)	✓	✓
	M66I	✓	✓	-
	I66V	-	✓[b]	✓
	V109A	✓	✓	✓
	T238N	-	-	✓
	C239G	✓	✓	✓
	V596I	✓ (blue)	✓ (blue)	✓
**PB1**	T156K	-	-	✓ (blue)[d]
	S261N	-	-	✓[e]
	N375D	✓[a]	✓	✓
	V664M	✓[a]	✓	✓
	V715M	✓	✓	✓
**PB1-F2**	E26G	-	-	✓
**PA**	E160K	✓ (blue)	✓	✓
	A231T	-	-	✓
	E237K	-	✓ (blue)[f]	✓
	A337T	✓[a]	✓	✓
	V432I	-	-	✓
	N614D	-	-	✓
**PA-X**	E160K	✓ (blue)	✓ (blue)	✓
	S193N	✓	✓ (blue)	✓
	P215L	-	-	✓
**HA**	A102V	✓	✓	✓
	E201K	✓ (blue)[a]	✓	✓ (blue)[g]
	S336N	-	-	✓
**NP**	K198R	✓ (blue)	✓ (blue)	✓
	M371I	-	✓ (blue)	✓
	T433P	-	-	✓ (blue)[h]
	N483K	-	-	✓
**NA**	I7T	-	-	✓
	I40R	-	-	✓
	N42I	-	-	✓
	I267V	-	✓ (blue)	✓
	V346I	-	✓ (blue)	✓
	S450G	-	-	✓
**M1**	K242N	-	✓ (blue)[b]	✓
**M2**	N13T	-	✓ (blue)[b]	✓
**NS1**	E71D	-	-	✓[e]
	V84M	-	✓ (blue)[c]	✓
	L90I	-	✓ (blue)[b]	✓
	S165P	✓ (blue)	✓	✓
**NEP**	none	-	-	-

Black tick: all viruses in SA13 contain the mutation; blue tick: some viruses in SA13 contain the mutation; -: no mutation.

[a]Also present in the seabird-origin viruses of SA4 (the progenitor to SA13)

[b]Present in a single virus from 2022, A/Common tern/South Africa/22060305/2022 (H5N1).

[c]Present in a single virus from 2022, A/African penguin/South Africa/702068 DOA370/2022 (H5N1).

[d]Present only in group C viruses

[e]Absent from A/Common tern/South Africa/CMT004/2023 *(me: more evidence this was the progenitor)*

[f]Present in a single virus from 2022, A/Common tern/South Africa/681998 CT10/2022

[g]Present only in group B1 viruses and A/Common tern/South Africa/CMT004/2023

[h]Present only in group B1 viruses
