# Supplementary figures and images for: Outbreaks of H5N1 High Pathogenicity Avian Influenza in South Africa in 2023 Were Caused by Two Distinct Sub-Genotypes of Clade 2.3.4.4b Viruses
Source: Viruses. 2024 May 31;16(6):896. doi: 10.3390/v16060896 (PMC11209199; doi:10.3390/v16060896)

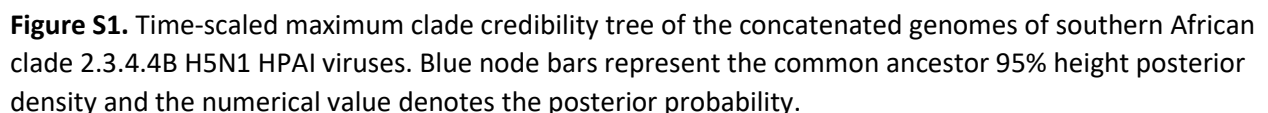

Supplement: Supplementary file 1 [file viruses-16-00896-s001.zip › viruses-3028589-supplementary materials/viruses-3028589-supplementary materials/Figure S1.pdf]
